# Investigation on the Performance of Coated Carbide Tool during Dry Turning of AISI 4340 Alloy Steel

**DOI:** 10.3390/ma16020668

**Published:** 2023-01-10

**Authors:** Naresh Kumar Wagri, Neelesh Kumar Jain, Anand Petare, Sudhansu Ranjan Das, Mohammed Y. Tharwan, Abdulkarim Alansari, Bader Alqahtani, Majed Fattouh, Ammar Elsheikh

**Affiliations:** 1 Department of Applied Physics and Electronics, Umeå University, 901 87 Umeå, Sweden; 2 Department of Mechanical Engineering, Indian Institute of Technology Indore, Indore 453552, India; 3 Department of Production Engineering, Veer Surendra Sai University of Technology, Burla 768018, India; 4 Mechanical Engineering Department, College of Engineering, Jazan University, KSA, 114 Almarefah Rd., Jazan 45142, Saudi Arabia; 5 Mechanical Engineering Department, College of Engineering, Northern Border University, Arar 91431, Saudi Arabia; 6 Department of Mechanical Engineering, Faculty of Engineering, University of Blue Nile, Damazeen 26613, Sudan; 7 Department of Production Engineering and Mechanical Design, Tanta University, Tanta 31527, Egypt

**Keywords:** chip morphology, cutting forces, coated tool, surface roughness, alloy steel

## Abstract

The machinability of materials is highly affected by their hardness, and it affects power consumption, cutting tool life as well as surface quality while machining the component. This work deals with machining of annealed AISI 4340 alloy steel using a coated carbide tool under a dry environment. The microhardness of annealed and non-annealed workpieces was compared and a significant reduction was found in the microhardness of annealed samples. Microstructure examination of the annealed sample revealed the formation of coarse pearlite which indicated a reduction of hardness and improved ductility. A commercially CVD multilayer (TiN/TiCN/Al_2_O_3_/ZrCN) coated cemented carbide cutting tool was employed for turning quenched and tempered structural AISI 4340 alloy steel by varying machining speed, rate of feed, and depth of cut to evaluate the surface quality, machining forces, flank wear, and chip morphology. According to the findings of experiments, the feed rate possesses a high impact on surface finish, followed by cutting speed. The prominent shape of the serrated saw tooth chip was noticed at a higher cutting speed. Machined surface finish and cutting forces during turning is a function of the wear profile of the coated carbide insert. This study proves that annealing is a low-cost and economical process to enhance the machinability of alloy steel.

## 1. Introduction

Alloy steel AISI (American Iron and Steel Institute) 4340 is nickel, chromium, and molybdenum-based commercial steel having high strength, and corrosion resistance. It is used for making flanges, gear shafts, gun components, gear, bearings, and components of aircraft such as landing gear and engine components such as pistons. The products made from AISI 4340 alloy steel have outstanding wear and longevity characteristics with proper work and reinforcement. Therefore, it is widely used for manufacturing components where long service life with high fatigue strength and good surface finish is required despite the surge in titanium-based alloys and composite materials [1,2,3]. Aerospace, automotive, machine tools, turbines, pumps, compressors, mining, defense, and construction are the major application areas of AISI 4340 alloy steel [4,5]. Annealing is the most frequently used process of heat treatment to modify the physical properties of materials by decreasing crystal dislocation and grain refinement. It enhances ductility, strength, toughness, fatigue life, and corrosion resistance without changing chemical composition. It causes grain refinement which reduces the hardness of the material and enhances the machinability of materials [6,7]. Annealing of AISI 4140 steel changes its microstructure by inducing a near-Masing-type effect at higher and lower strain amplitude which increases their ductility, plastic energy, and low-cycle fatigue strength [8,9]. Machinability of any metal is influenced by four-factors: (a) power, accuracy, and rigidity of the machine; (b) material and geometry of the cutting tool; (c) ductility, hardness, microstructure, and (d) production method of work materials [10,11,12]. Tool life, cutting forces, and surface finish are prime aspects while the hard turning of materials are important because it influences their wear characteristics, corrosion resistance, fatigue strength, and tribological performance of machined work [13,14]. Bouzid [15] conducted high-speed machining of AISI 4340 alloy steel employing a commercially coated cutting insert and reported that increments in cutting speed resulted in faster removal of tool coating, which caused an increase in tool wear rate. Aslan et al. [16] performed experiments aiming to attain minimal flank wear and surface roughness using several machining parameter combinations during the machining of hardened AISI 4140 steel by employing a ceramic cutting tool. As per the experimental results, both the machining responses decreased with increasing cutting speed and low feed rates. Asilturk and Akkus [17] investigated the role of machining parameters, namely axial feed, cutting depth and machining speed on the surface quality of the final product in turning AISI 4140 steel by employing a coated carbide tool. It was revealed that when speed and cutting depth increase, the surface roughness decreased. Secondly, when the machining was being conducted, employing the combination of high speed along with a lower doc and feed value, the surface finish improved. Coto et al. [18] assessed the effect of cutting parameters over residual stresses developed on machined surfaces in turning of AISI 4340 alloy steel. Saini et al. [19] used PVD and CVD-coated inserts under MQL-assisted turning and dry hard machining of AISI 4340 alloy steel. Results revealed that: (a) 17% reduction in cutting force using the CVD tool and 13% reduction in cutting force using the PVD insert, and (b) use of MQL reduces work tool interface temperature by 5.7% by CVD tool and 6.7% by PVD respectively. While performing experimental trials of a turning operation on hardened steel, Das et al. [20,21] concluded that the rate of feed followed by cutting speed was the influential variable for achieving the high surface finish. When the doc and cutting speed increased it resulted in higher flank wear. Sohrabpoor et al. [22] revealed that MQL machining improved the chip flushing and reduced the temperature at the machining zone which improved the surface quality and life of the tool. Higher cutting velocity, depth of cut, and coolant pressure with lower feed and cutting angle produced a low value of R_a_. At lower speed, feed and depth of cut along with high cutting angle and cutting fluid pressure improved the tool life. The consequence of machining variables over the surface quality of AISI 4340 alloy steel was studied by Agrawal et al. [23] and revealed that the surface quality of components was affected by doc and speed. Chinchanikar and Choudhury [24] evaluated tool life in hard turning, employing a coated carbide insert. As per the experimental observations with the increase in doc, the machining forces and interface temperature elevate which results in a decrease in tool life. Rashid et al. [25] analyzed the hard turning of AISI 4340 alloy steel with a CBN cutting insert and achieved a surface roughness of 0.045 µm. They reported that a low feed improves surface finish; however, it increases tool wear. Therefore, the feed rate must be selected considering the cost of production and the surface quality required. Khan and Bhivsane [26] analyzed the consequences of speed, nose radius, feed, and doc on machining of AISI 4340 alloy steel employing the CBN tool and found that speed and nose radius have a significant consequence on surface finish. Coated CBN tools have also been used to cut different alloys [27] such as AISI H11 steel (48–49 HRC) steel [28], AISI 4340 hardened steel [29], and alloyed steel [30]. Reis et al. [31] used a coated carbide cutting tool and a coated cermet cutting tool for turning AISI 4340 alloy steel to evaluate chip temperature, surface finish, and tool wear. They reported that (a) coated cermet tool produced a higher chip temperature and more flank wear compared to the coated carbide tool, and (b) the coated carbide tool resulted in more surface finish compared to the coated cermet tool. Raof et al. [6] performed the machining of quenched followed by tempered HSLA steel under dry and wet environments using the coated carbide insert. They revealed that wet turning causes a reduction in temperature at the machining zone, thus resulting in improvement in surface quality. Wet turning causes the formation of ultrafine white globular particles on a work surface which enhances their strength and hardness compared to dry turning. Three different tools, namely ceramic tools (class CC650 and CC6050) and polycrystalline boron nitride (PCBN) were used by Da-Silva et al. [32] to evaluate the turned finished surfaces of AISI 4340 alloy steel. The results revealed that the ceramic tool CC6050 gives a more finished surface followed by the PCBN tool and last by the ceramic CC650 tool for both dry as well as wet turning.

The conclusion of this literature survey is that previous research work has mostly focused on evaluating tool wear and surface finish while machining AISI 4340 alloy steel. Very limited work has been in the direction of analysis of chip morphology and cutting forces during AISI 4340 alloy steel machining. No work has been reported to enhance the machinability of AISI 4340 alloy steel keeping a lower cost of production and a high-quality surface finish. The capabilities of the annealing process to improve ductility and reduce the hardness of AISI 4340 to enhance machinability have not been explored. Although limited research has been published, there are experimental reports on the machinability of AISI 4340 alloy steel with expensive ceramic and CBN cutting insert for the reason of prolonging cutting tool life. The available experimental literature lacks the desired information, which reports the machining performance of inexpensive coated carbide tools in turning, which is considered one of the important research areas from an economical point of view. While keeping the contributions made by previous research, the present article deals with the machinability investigation of annealed AISI 4340 alloy steel in turning using coated cemented carbide inserts under a dry cutting atmosphere for low production cost and high surface finish. Several machining temperaments like tool flank wear, cutting forces, chip morphology, and surface roughness are addressed under the varying process variables like depth of cut, feed, and speed. A novelty aspect of the present work is that it proposes an alternative to costlier ceramic and CBN tools by utilizing coated carbide tools in dry turning processes, considering techno-economical and ecological aspects which are helpful for the shaft and die makers in practical industrial applications.

## 2. Materials and Methods

### 2.1. Workpiece Material

**Figure 1:**
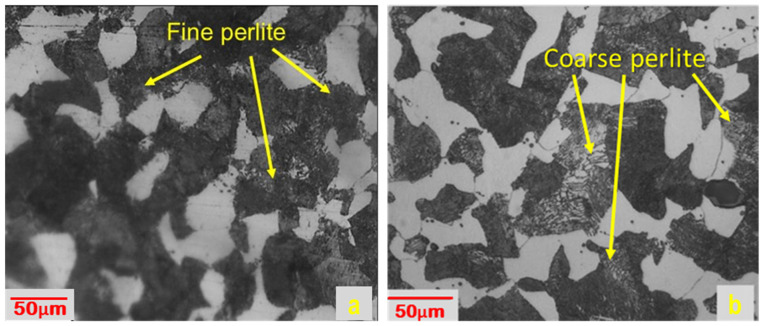
Optical micrograph of AISI 4340 alloy steel: (**a**) Before; and (**b**) After heat treatment.

A cylindrical-shaped 50 mm dia and 150 mm long AISI 4340 alloy steel was used as the workpiece materials for the machining operation. It poses a Rockwell hardness value of 17 on C-scale (HRC) and chemical composition (by weight percentage) as 0.37% C; 1.65% Ni; 0.7% Cr; 0.6% Mn; 0.2% Mo; 0.15% Si; 0.04% S; 0.035% P and the rest of the balance Fe. For heat treatment (i.e., annealing) of the workpiece material an induction furnace Inductoheat 50-50R (from Inducotherm, Sanand, India) was used, having a maximum working temperature of 1200 °C. The workpiece samples were heated at 500 °C for 2 h and then left in the furnace to cool for 24 h, to increase ductility and made them soft to enhance machinability. A German-made Vicker’s microhardness tester (model: VMH-002 make: Walter UHL) was employed to determine the microhardness value before and after the annealing of workpiece samples by applying a force of exactly 200 g for a time span of 15 s as per the procedure prescribed in ASTM E92-82 [33]. Three indentations were made and their average value was used for the investigation. The average microhardness before heat treatment was 263 HV, the same has been reduced to 241 HV after the heat treatment. Figure 1 presents optical micrographs, showing the surface morphology of the workpiece sample before annealing Figure 1a and after heat annealing Figure 1b. It can be seen from the workpiece sample that the sample before heat treatment contained a fine perlite structure in which fine layers were present which opposes relative slipping to each other and hence higher hardness. The fine perlite structures were converted to coarse perlite structures after heat treatment which causes grain refinement, reduces hardness, and increases ductility [34].

### 2.2. Details of Experimentation

**Figure 2:**
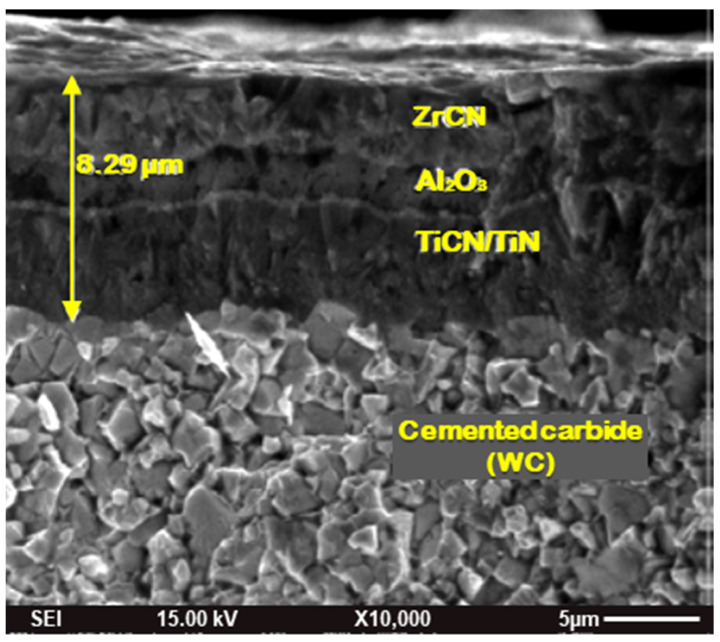
Cross-section morphology of coated cemented carbide tool.

Turning experiments were performed on lathe machine NH-22 (from Hindustan Machine Tools, Bangalore, India) having a speed range of 40 to 2040 rpm and longitudinal feed range from 0.04 to 2.24 mm/rev. In the present investigation, a CVD multilayer (TiN/TiCN/Al_2_O_3_/ZrCN) coated cemented carbide cutting insert with ISO designation of SCMT120408 and grade of KCP30 (manufactured by Kennametal, Pittsburgh, PA, USA) was used for the turning operation. The cutting insert was mounted on a tool holder with an ISO designation of SSBCR2020K12. The combination of the cutting insert with the tool insert holder resulted in the cutting geometry of −6° - −6° - 6° - 6° - 15° - 75° - 0.8 (i.e., −6° back rake, −6° side rake angle, 6° end clearance angle, 6° side clearance angle, 15° end cutting edge angle, 75° approach angle and 0.8 mm nose radius). Figure 2 displays an SEM image of the cross-section morphology of coated carbide tool, showing the various coating layers on cemented carbide tool substrate. Some preliminary turning experiments were performed on workpiece materials by varying cutting speeds (6, 8, 11, 16, 23, 27, 30, 39, 66, 77, 86, 112, 146, 190, 247, and 320 m/min) as per the standard speed available on the lathe machine using doc at 0.1 mm and feed at 0.1 mm/rev. The wear behavior of the coated carbide tool was assessed on the basis of allowable flank wear (VB_max_) limits of VB up to 0.3 mm (as per ISO 3685 i.e., maximum flank wear width occurring at the tip of the tool). To derive the interaction, which governs the cutting forces and machined surface roughness, it is insightful to correlate with the resulting wear behavior. Based on these experiments, a cutting speed ranging from 112 to 247 m/min was found to be an optimum cutting speed for further experiments. Using the findings of the preliminary experiments, 16 experiments were designed and conducted using the Taguchi L_16_ technique by varying the three most important parameters at four levels. The various levels of selected machining variables in these experiments are listed in Table 1. All experiments were performed with a new cutting edge and the machining was continued for the duration of 10 s.

#### 2.2.1. Surface Roughness Evaluation

Average surface roughness (*R_a_*), surface roughness depth (*R_z_),* and maximum surface roughness (*R_max_*) were evaluated using a MarSurf LD-130 roughness tester as per ISO-4287. Considering the 4 mm evaluation length and 0.8 mm cut-off length two traces at two different places of the workpiece were taken using a ϕ10 µm size stylus. Thereafter, arithmetic means of all the measured values of ‘*R_a_*’, ‘*R_max_*’, and ‘*R_z_*’ were computed for further analysis.

#### 2.2.2. Evaluation of Cutting Forces

Cutting forces play a vital role during the cutting process and are a cause of extensive stresses and plastic deformations in the material. The cutting force created by the turning process resolves into three components cutting force (*F_z_),* feed force (*F_x_)_,_* and radial force (*F_y_)*. A piezoelectric dynamometer 5070A (from Kistler, Winterthur, Switzerland) was used to evaluate cutting forces as per ISO 841. It has Dynoware software with a data acquisition system that converts the signal received from multichannel and coverts it into a graphical format to display over the monitor. A measuring time of 10 sec and a sampling rate of 1000 Hz was used to measure cutting forces and each experiment was repeated thrice and their mean values were considered for further evaluation.

#### 2.2.3. Evaluation of Generated Chips

**Figure 3:**
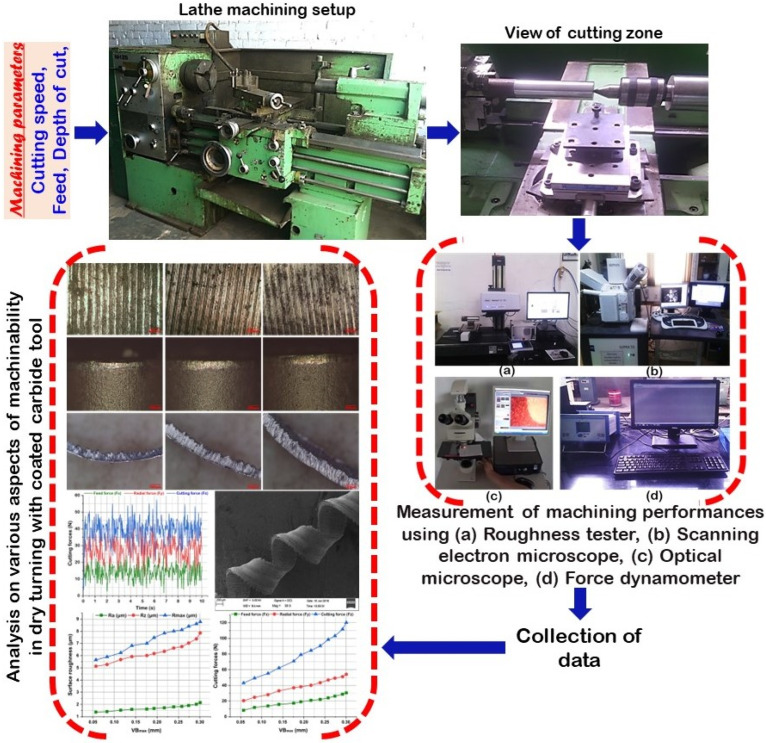
The layout of the experimental setup and procedure.

Analysis of developed chips was made by using Supra 55 scanning electron microscopic (SEM) from Carl-Zeiss NTS GmbH, Oberkochen, Germany to examine chip morphology. The average chip thickness was measured with the help of a digital caliper (make: Mitotoyo) by considering an average of fifteen samples from each experimental test. Consequently, the chip thickness was confirmed with an optical microscope (make: Leica, model: DM2500 M) for selected chips. Figure 3 shows a detailed schematic of the experimental setup and procedure.

## 3. Results and Discussion

The different levels of variable parameters and the values of responses obtained for each experiment are detailed in Table 2. It is noted that hard machining under V = 146 m/min, d = 0.1 mm, f = 0.10 mm/rev (corresponding to experiment number 5) produced minimum values in average surface roughness ‘*R_a_*’ (0.73 µm), in maximum surface roughness ‘*R_max_*’ (4.17 µm) and surface roughness depth ‘*R_z_*’ (3.98 µm). Similarly, hard turning under V = 112 m/min, d = 0.10 mm, f = 0.1 mm/rev produced minimum values in feed force ‘*F_x_*’ (2.55 N), in radial force ‘*F_y_*’ (8.64 N), in tangential force ‘*F_z_*’ (15.98 N) and maximum flank wear ‘*VB_max_*’ (0.01 mm), corresponding to experiment number 1. The machining parameter settings at V = 146 m/min, d = 0.1 mm, f = 0.16 mm/rev (corresponding to experiment number 6) produced a minimum value in chip thickness ‘*t_c_*’ (0.14 mm).

**Figure 4:**
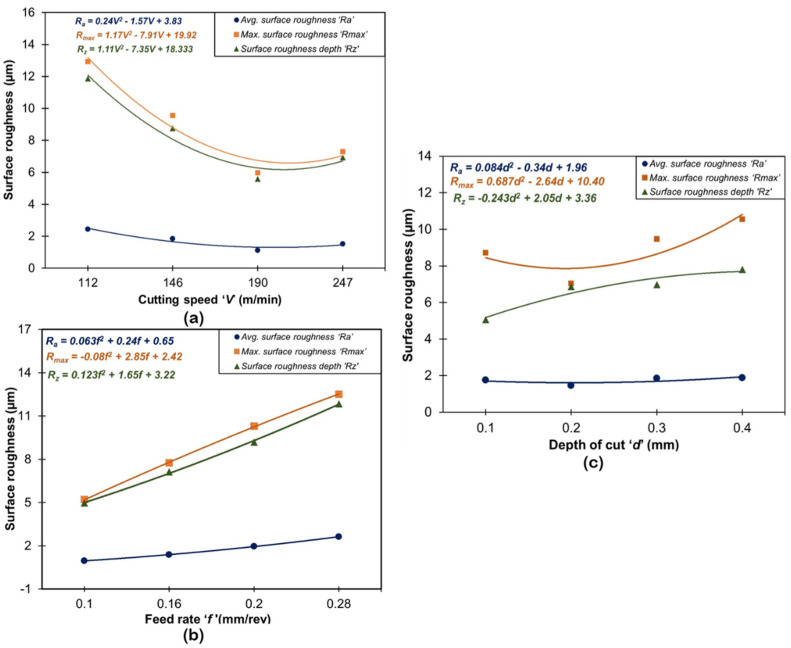
Variation in surface roughness parameters under the influence of: (**a**) speed; (**b**) feed; (**c**) depth of cut.

**Figure 5:**
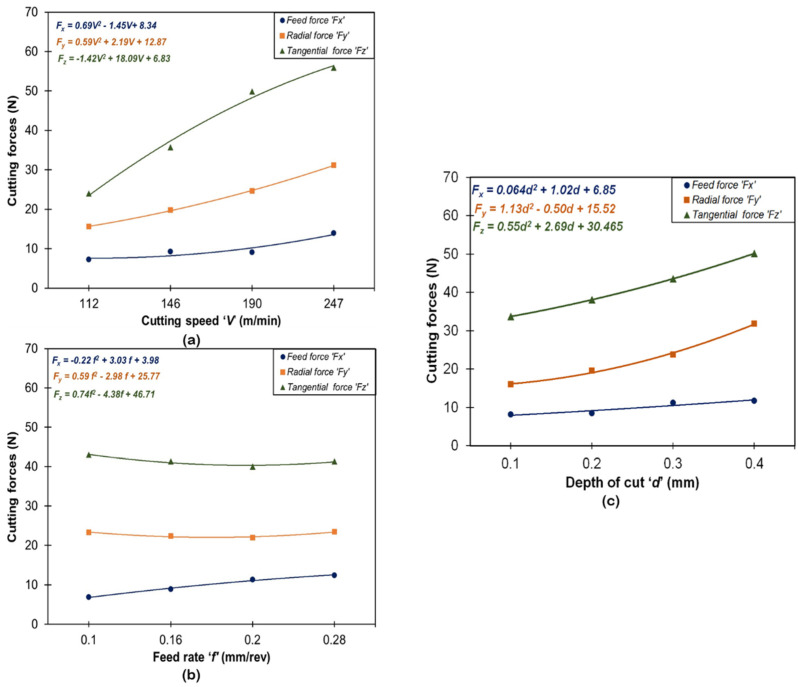
Variation in cutting forces under the influence of: (**a**) speed; (**b**) feed; (**c**) depth of cut.

Figure 4 and Figure 5 show the impact of machining factors (V, f, d) on surface quality parameters (R_a_, R_max_, R_z_) and machining forces (F_x_, F_y_, F_z_). Regression analyses were employed to obtain the predictive models for each response on experimental values and their curve fitting. ANOVA was used to test the significance of the predictive models by using MINITAB 20 software. The probability value (i.e., the *p*-value) corresponding to each equation was found below 0.05 which indicated the statistical significance of experimental data at a 95% confidence level. The results of ANOVA are provided in Table 3.

To establish the relationship between cutting regime parameters (V, f, d) and technological characteristics of machinability (R_a_, R_max_, R_z_, F_x_, F_y_, F_z_), the results of several machining trials conducted as per L_16_ OA experimental design were analyzed by employing multifactorial method. The developed predictive models are provided in the form of a non-linear regression Equations (1)–(6) as,

(1) $$R_{a}=573.785\;V^{-0.822705}f^{1.00517}d^{-0.0497773}\;(R^{2}\;=\;0.953)$$

(2) $$R_{max}=3857.21\;V^{-0.911301}f^{0.792965}d^{0.0632166}\;(R^{2}\;=\;0.973)$$

(3) $$R_{z}=3150.99\;V^{-0.891816}f^{0.841068}d^{-0.00885609}\;(R^{2}\;=\;0.964)$$

(4) $$F_{x}=0.608758\;V^{0.862885}f^{0.625934}d^{0.394659}\;(R^{2}\;=\;0.926)$$

(5) $$F_{y}=0.650214\;V^{0.869776}f^{0.0909116}d^{0.530625}\;(R^{2}\;=\;0.977)$$

(6) $$F_{z}=0.45889\;V^{0.958169}f^{0.0151052}d^{0.277836}\;(R^{2}\;=\;0.991)$$

The proposed regression models may be employed to predict various machinability performances due to their high R^2^ (coefficient of determination) value, which approached a value of 1 and confirmed the excellent goodness-of-fit of the model. In our future work we will apply advanced machine learning approaches to accomplish this task [35]. 

**Figure 6:**
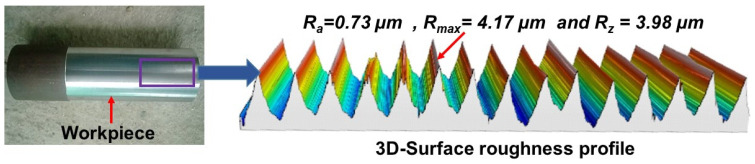
3-D profile of best finished surface of AISI 4340 alloy steel workpieces obtained at V = 146 m/min, d = 0.4 mm, f = 0.1 mm/rev.

From Figure 4 and Table 3, feed rate was found to be one of the most influential parameters contributing 51.8% for *R_a_*, 42.7% for *R_max_*, and 44.3% for *R_z_*. This result is anticipated with the very well-known theory of metal cutting that for a specified or stated nose radius, the theoretical surface roughness (*Ra* = 0.032$\frac{f^{2}}{r_{e}}$) is principally a function of feed rate [36,37]. The second most influencing parameter was observed in cutting speed with a contribution of 51.8% for *R_a_*, 39.6% for *R_max,_* and 37.8% for *R_z_.* Although, the parameter cutting speed does not present a statistically significant role on surface roughness (especially, Ra) because its *p*-value is larger than 0.05; however, it is a lone contribution (30.93%) and cannot be ignored. This is in good agreement with previously published works [38,39,40]. The error contributions have shown a very minor impact on roughness parameters (*R_a_*, *R_max,_* and *R_z,_*) with 13.4%, 8.5%, and 11.1%, respectively. These small values indicate no errors in the measurement of responses. Increasing the cutting speed, surface roughness parameters starts decreasing and attains a minimum value at a speed of 190 m/min and thereafter surface roughness starts increasing. At a lower cutting speed, a built-up edge (BUE) developed which sticks over the carbide insert and results in more surface roughness; whereas as soon as the cutting speed starts increasing (up to 190 m/min), the temperature at the chip-tool interface and the time required for welding of chip material over the tool is inadequate. This inhibits the formation of the build-up-edge and results in a decrease in surface roughness [41]. Surface roughness parameters start slightly increasing the cutting speed beyond 190 m/min due to the worn cutting insert. Two major factors (lateral side flow arising from plastic deformation of machined surface and chatter due to vibration) influenced the roughness at a high level of cutting speed (247 m/min), as reported by Mahapatra et al. [42]. In the case of increasing feed rate on all surfaces, roughness variables start increasing and attain high values at a feed of 0.28 mm/rev. At a low value of feed, the formation rate of BUE and tool wear was less, and as the feed increases BUE formation and cutter feed marks increase, which results in more surface roughness. In addition, Suresh et al. [43] reported that with the increased axial feed, the thrust force increases accordingly, provoking unusual vibration and high heat generation that corresponds to poor surface finish. Similarly, the increase in depth of cut causes the 0.1 to 0.2 mm surface roughness parameters to decrease; thereafter, they start increasing and attain the highest value at 0.4 mm. This is due to an increase in cutting depth which causes more material removal from work materials, high heat generation at the tool-chip tool, and generation of feed (cutter) marks. Figure 6 depicts the 3-D profile of the best-finished surface workpiece sample.

**Figure 7:**
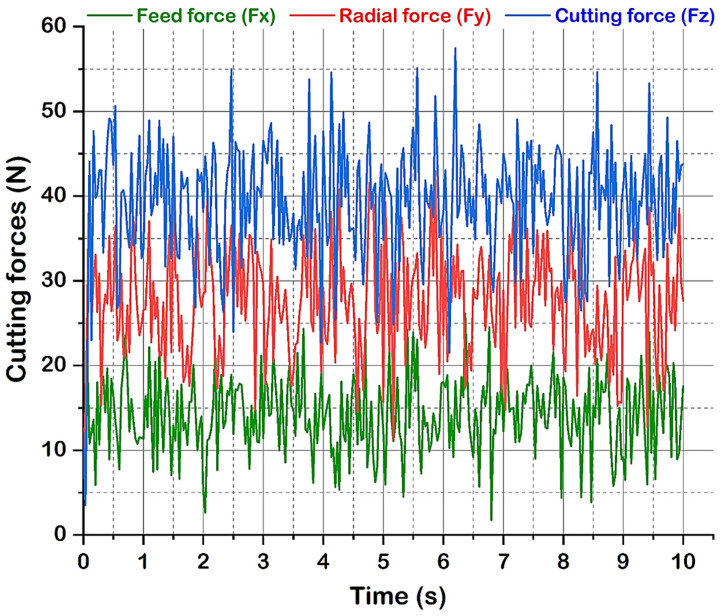
Cutting forces (F_x_, F_y_, F_z_) at experimental run order 4 (V = 112 m/min, f = 0.28 mm/rev, d = 0.40 mm).

Cutting speed is the most significant parameter contributing 40% for *F_x_*, 47.8% for *F_y,_* and 78% for *F_z_*. The next contributing significant parameter was the depth of cut, recorded at merely 50% for *F_y_* and 19.2% for *F_z_*. The depth of cut had a very minor effect on axial force or feed force (*F_x_*) because this force is directed parallel to the center line of the workpiece [44,45]. Moreover, the feed rate showed a very minor impact and was identified as statistically insignificant on cutting forces, especially for *F_y_* and *F_z_*, whereas the feed rate was significant at 30.6% only for feed force (*F_x_*). Therefore, the error contributed 12.4%, 1.5%, and 2.1% for *F_x_*, *F_y,_* and *F_z_*, respectively. All cutting forces increase with cutting speed and doc and attain a maximum value at 247 m/min and 0.4 mm, as shown in Figure 5. Hard turning under high cutting speed with high feed and doc causes more material removal, more BUE formation, and an increase in heat generation which affects tool life and increases cutting forces. Figure 5a shows a significant increase in cutting force with the increase in cutting speed, which contradicts the common expectation that cutting forces usually decrease with increasing cutting speed. This outcome can be explained by the predominance of the strain hardening effect over the thermal softening process at elevated temperatures. In addition to this, the cutting tool inserts have a square geometry shape with a principal cutting angle of 75° and a point angle of 90° which creates a larger contact area between the tool and work with less time. Hence, the excess amount of material removal requires more plastic deformation by increasing the cutting forces. A proper explanation has been reported regarding cutting forces variation at increased speeds, as explained by Das et al. [46]. Figure 5b,c indicates that as the feed rate and depth of cut increase, the cutting components also increase. However, the increased feed rate increases cutting forces marginally. As the feed rate and depth of cut are increased, the tool-chip interface area also increases, and the region of sheared chip increases, since resistance to material rupture is higher and hence requires larger efforts for chip removal [47]. The increased feed and depth of cut induced a larger volume of cut material in the same unit of time, in addition to establishing a dynamic effect on cutting forces. This in turn leads to a corresponding increase in normal contact stress at the tool chip interface and in the tool chip contact area and hence the cutting forces have increased. Figure 7 depicts the signals of cutting forces obtained at a speed of 112 m/min, a feed of 0.28 mm/rev, and a cutting depth of 0.4 mm, corresponding to experiment number 4.

**Figure 8:**
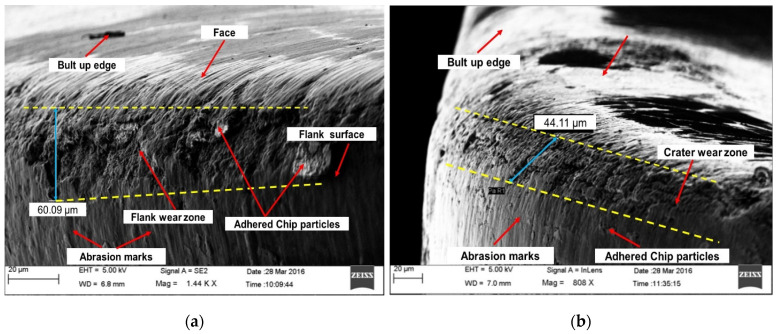
SEM images of a worn-out insert illustrating: (**a**) flank wear, (**b**) crater wear.

**Figure 9:**
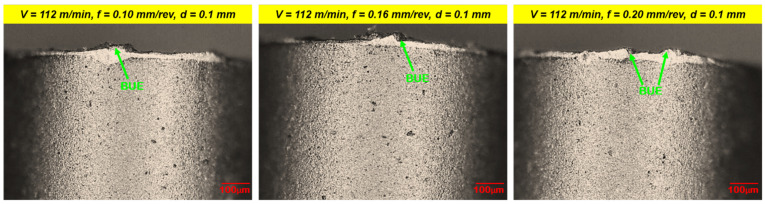
SEM images of a worn-out insert illustrating built-up-edge (BUE) formation.

Figure 8 depicts a surface micrograph of a worn-out insert of a tool at a cutting speed of 247 m/min, feed rate of 0.1 mm/rev, and depth of cut of 0.4 mm for flank wear. It can be noted from (i) Figure 8a that the flank face, rack surface, and nose of the insert are severely worn at high speed and doc. The maximum flank wear was 60.09 µm. The presence of deep plowing grooves and edges on the flank and crater surface indicates the abrasion mode of wear of the insert [21]. Chip particles also adhered to the flank and crater surface which causes nose wear due to the welding of chips; and (ii) Figure 8b that maximum crater wear of 44.11 µm was found by SEM. Increasing the depth of cut causes more material to be removed from the work surface as it passes over the rack face of the tool, and it causes the removal of material from the rack surface because of its high temperature. This results in crater wear and causes flank wear. It is evident from Figure 9 that at low cutting speeds develop built-up-edge formation to a great extent which may be due to sufficient available time for microchips to adhere to the tool face through the welding effect (developed by friction and heat at the tool-chip interface) [48]. Additionally, Ahmed et al. [49] reported that a low thermal conductivity and high work hardening tendency of work materials during machining are also considerations for BUE formation.

**Figure 10:**
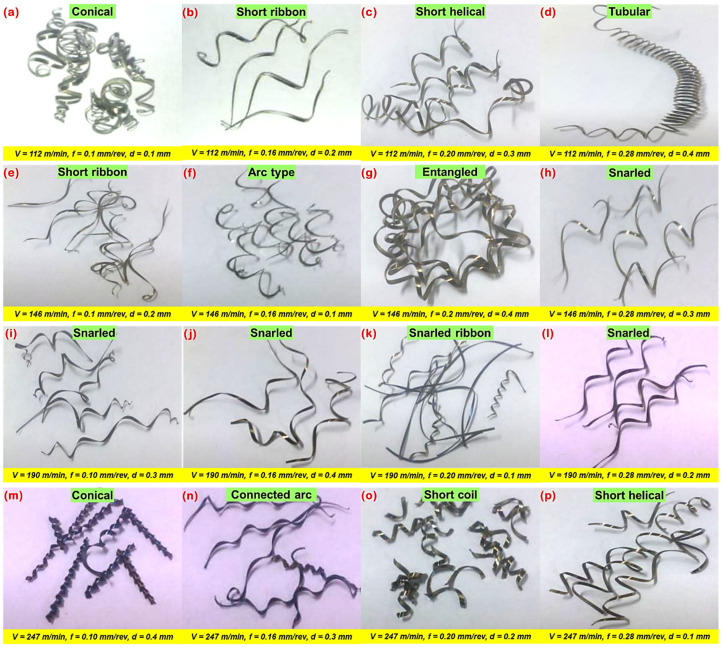
Photograph of chips at various feed rates and cutting speeds.

After performing a series of cylindrical turning trials under various cutting conditions, the produced chips were collected and categorized as per ISO 3685 standard for quantitate evaluation. The nature of the chips produced during machining highly influences the machining quality and machinability aspects. Figure 10 represents the various size and shapes of chips produced from the machining operations. Chip thickness increases for every value of speed, doc, and feed. However, it attains the maximum value at a speed of 247 m/min, an axial feed of 0.28 mm/rev, a cutting depth of 0.4 mm. Figure 10 depicts photographs of chips obtained under several cutting speeds and feeds. It is seen that lower chip thickness appears to decrease with feed and speed, which contrarily decreases the cutting forces as well as tool vibration and, thereby, results in better surface quality. Undeformed chip thickness increases with feed, as stated. The generation of the saw-tooth chips depends directly on the undeformed chip thickness; in particular, an increase in the thickness of the undeformed chip contributes to a larger saw-tooth.

**Figure 11:**
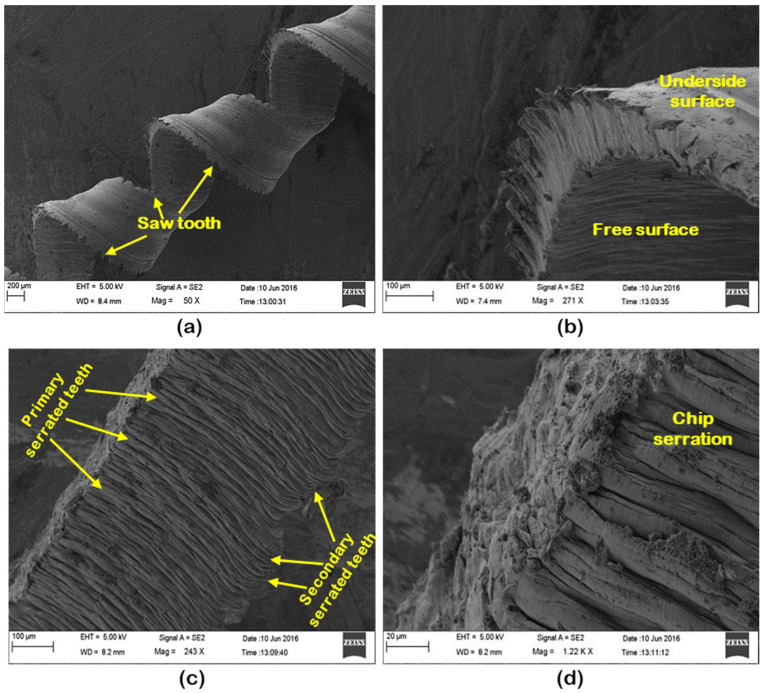

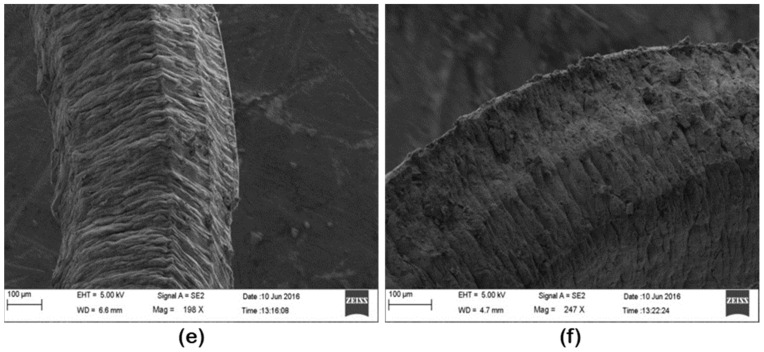
Chip morphology at: (**a**) V = 247 m/min, d = 0.4 mm, f = 0.10 mm/rev; (**b**) V = 247 m/min, d = 0.1 mm, f = 0.28 mm/rev; (**c**) V = 170 m/min, d = 0.3 mm, f = 0.10 mm/rev; (**d**) V = 146 m/min, d = 0.2 mm, f = 0.10 mm/rev; (**e**) top view of chip; and (**f**) bottom view chip at V = 146 m/min, d = 0.2 mm, f = 0.10 mm/rev.

Figure 11 depicts the chip morphology of AISI 4340 alloy steel produced by dry turning under various machining settings (conditions). Serrated chips are noticed in all the cutting conditions. Plastic deformation, cyclic cracks, and shear localization are the three primary attributes of chip serration. It can be noted from Figure 11a,b that the chips were gradually scalloped with the increase of axial feed and doc at a cutting speed of 247 m/min. Such implies that the saw tooth chip takes more and more form as a result of continuous cyclic cracking produced by intense shear bands. Both top and bottom surfaces of the chip were confirmed with SEM observation. Plastic deformation is marked at the top surface of the chip, which resulted in a continuous rough surface mostly with ridges and scallops. In the case of the underside surface of the chip, which was sliding upon the tool, it is considerably smoother with fine-long scratches. It is seen from Figure 11c,d that the chip serrations are moving above the whole width of the chip at varying machining conditions. Distinct saw tooth elements have appeared over the upper-free edge, which is marked as primary serrated teeth, and in certain instances, bigger coagulated features developed at the lower-tool tip-side edge of the chip, which is marked as secondary serrated teeth. The free (upper) surface of the cutting chips produced under cutting conditions; v = 146 m/min, f = 0.10 mm/rev, and d = 0.2 mm, is shown in Figure 11e,f. The SEM image depicts the severity of chip serration, including the appearance of the rough surface and irregularly arranged lamellar structure with minute corrugations. This is due to lateral flow and shear cracks that initiate at the free surface of the workpiece and proceed downward along a shear plane towards the cutting edge and subsequently to the rake surface. Based on the above result and discussion, the combination of speed at 146 m/min, axial feed as 0.1 mm/rev, and doc as 0.4 mm is recognized as an optimum parametric combination merger that yields a good surface finish (in experiment no. 5), whereas the combination of V = 112 m/min, d = 0.10 mm, f = 0.10 mm/rev is identified as an optimum combination which yields low cutting forces and minimum flank wear. However, the parametric combination of V = 146 m/min, d = 0.10 mm, f = 0.16 mm/rev is identified as the optimum condition for the production of low chip thickness.

**Figure 12:**
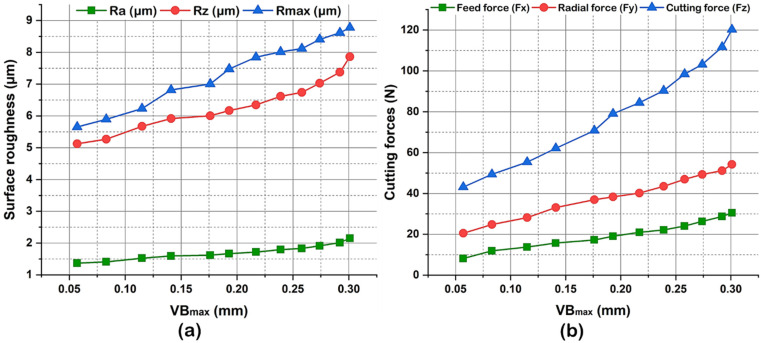
Influence of tool flank wear on (**a**) surface roughness, and (**b**) Cutting forces at V = 247 m/min, f = 0.28 mm/rev, d = 0.4 mm.

**Figure 13:**
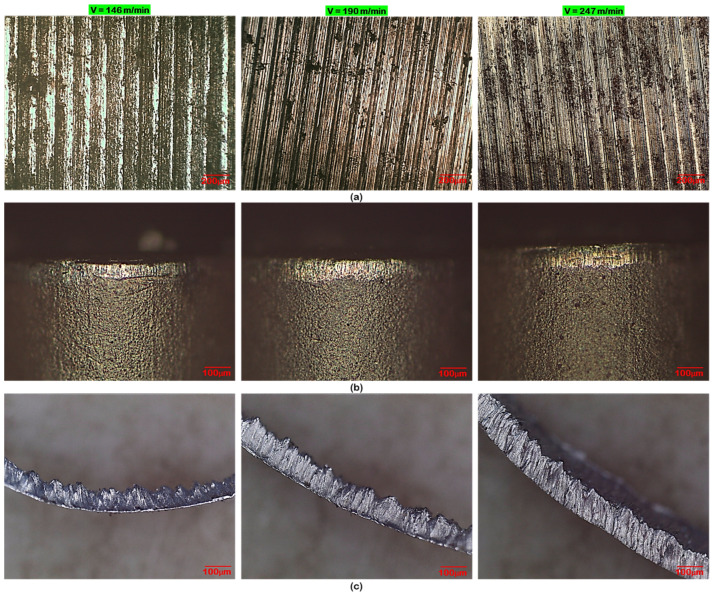
Machining performance assessment under different speeds concerning: (**a**) machined surface morphology, (**b**) tool flank wear, and (**c**) chip thickness.

Figure 12 illustrates the influence of tool flank wear on machined surface finish and cutting forces. It is recorded from Figure 12a that a flank wear (VB_max_) value of 0.057 mm corresponds to the values of surface roughness criteria Ra, Rz and Rmax of 1.371, 5.122, and 5.652 µm, respectively and the values of machining force components (F_x_, F_y_, F_z_) are 43.15 N, 20.57 N and 8.17 N, respectively (refer, Figure 12b). When the flank wear attains 0.3 mm, the increase of surface roughness criteria Ra, Rz and Rmax is 57.18%, 53.49%, and 55.25%, respectively. The analysis concludes that surface roughness is closely related to and proportional to flank wear. That means any progress in flank wear indicates some degradation of the machined surface quality. Similar results can be found in the literature [20,50]. Consequently, when the flank wear reaches the value of 0.3 mm, a total rise in the cutting force components of 273.81%, 163.58%, and 178.72% was recorded for feed force, radial force, and cutting (tangential) force, respectively. The major cutting force is the tangential force. This synthesis confirms that the increase in cutting forces is in direct connection with the degradation of the cutting edge of the tool. Figure 13 illustrates the various machinability characteristics for the evaluation of machining performance under varying cutting speeds assessed in this study. It is seen that the enhanced machined surface morphology, higher chip thickness, developed prominent serrated chip, and increased tool wear were obtained when the machining was performed under a dry environment at a high cutting speed.

## 4. Conclusions

This paper has reported on the machining of an annealed AISI 4340 alloy steel via coated cemented carbide tool under a dry environment. The following outcomes can be drawn from this research:

- Annealing resulted from the lowering of machining forces and surface roughness by changing a fine perlite structure into a coarse perlite structure, which reduced the hardness of AISI 4340 alloy steel.
- Feed was noted as the most influential cutting variable on the surface roughness of the machined components. The cutting speed was found to be the second most influential parameter influencing the machined surface quality. Whereas depth of cut shows a very minimal impact on the surface finish of machined components. Increasing feed increases the formation of BUE and cutter feed marks which deteriorates the surface finish. An increase in machining speed causes the restriction of BUE formation and it results in an improvement in surface finish. A high depth of cut results in more material removal and more heat generation, which deteriorates the surface finish.
- Machining under a higher range of speed, feed and doc resulted in more material removal from the workpiece surface in a short period which resulted in a raising of cutting forces. High speed and doc cause intense heat generation at the work-tool interface and rubbing of the tool takes place which results in extreme flank wear. High-value depth of cut results in high chip thickness but it deteriorates surface finish because of high cutting forces and excessive tool vibration.
- Microscopic images of worn-out tools show the presence of BUE and adhered chip particles. The existence of marks on the bottom of the flank surface suggests an abrasion mode of wear. SEM observation of chips demonstrates the generation of saw-tooth chips with even shear patterns.
- Machined surface finish and cutting forces in turning is a function of the wear profile of coated carbide inserts.
- The above study proves annealing is a simple, easy, and economic process to improve the machinability of AISI 4340 alloy steel. This study will be helpful for industries and tool rooms that are involved in the machining of alloy steel for manufacturing components.

## Figures and Tables

**Table 1:** Cutting variables and levels.

Input Parameters	Levels			
	I	II	III	IV
Cutting speed ‘V’ (m/min)	112	146	190	247
Feed ‘f’ (mm/rev)	0.10	0.16	0.20	0.28
Depth of cut ‘d’ (mm)	0.1	0.2	0.3	0.4
Fixed input parameters: Machining time: 10 s; Temperature: 31 °C				

**Table 2:** Summary of input variables and responses obtained for each experiment.

Exp. No.	Variable Parameters			Machining Responses						
	V (m/min)	f (mm/rev)	d (mm)	R_a_ (µm)	R_max_ (µm)	R_z_ (µm)	F_x_ (N)	F_y_ (N)	F_z_ (N)	t_c_ (mm)
1	112	0.10	0.1	1.58	7.92	7.54	2.55	8.64	15.98	0.15
2	112	0.16	0.2	2.41	12.00	11.60	4.36	12.90	17.28	0.25
3	112	0.20	0.3	2.42	13.90	12.00	9.82	15.23	27.53	0.24
4	112	0.28	0.4	3.35	17.90	16.30	12.18	25.67	35.31	0.33
5	146	0.10	0.2	0.73	4.17	3.98	4.85	15.85	37.45	0.16
6	146	0.16	0.1	1.11	6.61	6.14	6.90	13.54	29.94	0.14
7	146	0.20	0.4	2.32	13.10	11.30	10.69	27.90	39.24	0.34
8	146	0.28	0.3	3.27	14.20	13.60	14.66	21.82	36.39	0.25
9	190	0.10	0.3	0.75	4.37	4.15	5.25	26.14	52.52	0.19
10	190	0.16	0.4	1.05	6.80	5.75	9.18	31.22	60.15	0.17
11	190	0.20	0.1	1.56	7.65	7.37	11.19	18.31	41.13	0.26
12	190	0.28	0.2	1.12	5.27	5.10	10.89	22.84	45.64	0.30
13	247	0.10	0.4	0.81	4.37	4.21	14.89	42.67	65.93	0.23
14	247	0.16	0.3	0.96	5.61	5.03	15.14	31.89	57.83	0.20
15	247	0.20	0.2	1.55	6.53	6.12	13.65	26.57	51.97	0.28
16	247	0.28	0.1	2.78	12.70	12.40	12.07	23.49	47.83	0.31

**Table 3:** Results of ANOVA for average surface roughness (R_a_), maximum surface roughness (R_max_), surface roughness depth (R_z_), cutting force (F_x_), feed force (F_y_), radial force (F_z_), flank wear (VB_max_).

Sources	*DOF*	*SS*	*MS*	*F*	*P*	Contr. %	Remark
(a) For average surface roughness ‘*R_a_*’							
Cutting speed ‘*V*’	3	3.7410	1.2470	4.61	0.053	30.926	
Feed rate ‘*f*’	3	6.2705	2.0902	7.73	0.017	51.842	Significant
Depth of cut ‘*d*’	3	0.4620	0.1540	0.57	0.655	3.819	
Error	6	1.6217	0.2703			13.407	
Total	15	12.0952				100	
(b) For maximum surface roughness ‘*R_max_*’							
Cutting speed ‘*V*’	3	111.218	37.073	9.32	0.011	39.636	Significant
Feed rate ‘*f*’	3	119.881	39.960	10.04	0.009	42.729	Significant
Depth of cut ‘*d*’	3	25.991	8.664	2.18	0.192	9.262	
Error	6	23.869	3.978			8.506	
Total	15	280.959				100	
(c) For surface roughness depth ‘*R_z_*’							
Cutting speed ‘*V*’	3	88.240	29.413	6.77	0.024	37.808	Significant
Feed rate ‘*f*’	3	103.460	34.487	7.94	0.016	44.329	Significant
Depth of cut ‘*d*’	3	15.629	5.210	1.20	0.387	6.696	
Error	6	26.056	4.343			11.164	
Total	15	233.386				100	
(d) For feed force ‘*F_x_*’							
Cutting speed ‘*V*’	3	97.600	32.533	6.42	0.027	40.082	Significant
Feed rate ‘*f*’	3	74.636	24.879	4.91	0.047	30.651	Significant
Depth of cut ‘*d*’	3	40.835	13.612	2.68	0.140	16.770	
Error	6	30.424	5.071			12.494	
Total	15	243.495				100	
(e) For radial force ‘*F_y_*’							
Cutting speed ‘*V*’	3	535.86	178.62	62.02	0.000	47.877	Significant
Feed rate ‘*f*’	3	6.06	2.02	0.70	0.585	0.541	
Depth of cut ‘*d*’	3	560.02	186.67	64.82	0.000	50.036	Significant
Error	6	17.28	2.88			1.543	
Total	15	1119.22				100	
(f) For cutting force ‘*F_z_*’							
Cutting speed ‘*V*’	3	2461.11	820.37	73.05	0.000	78.083	Significant
Feed rate ‘*f*’	3	18.15	6.05	0.54	0.673	0.575	
Depth of cut ‘*d*’	3	605.27	201.76	17.96	0.002	19.203	Significant
Error	6	67.38	11.23			2.137	
Total	15	3151.91				100	
